# Sodium hydrosulfide induces systemic thermotolerance to strawberry plants through transcriptional regulation of heat shock proteins and aquaporin

**DOI:** 10.1186/1471-2229-14-42

**Published:** 2014-02-05

**Authors:** Anastasis Christou, Panagiota Filippou, George A Manganaris, Vasileios Fotopoulos

**Affiliations:** 1 Department of Environmental Science and Technology, Cyprus University of Technology, 3603 Lemesos, Cyprus; 2 Department of Agricultural Sciences, Biotechnology and Food Science, Cyprus University of Technology, 3603 Lemesos, Cyprus; 3 Present address: Agricultural Research Institute, 1516 Nicosia, Cyprus

**Keywords:** Ascorbic acid, Heat shock proteins, Hydrogen sulfide, Nitrosative stress, Oxidative stress, Priming, Thermotolerance, *Fragaria* x *ananassa*

## Abstract

**Background:**

Temperature extremes represent an important limiting factor to plant growth and productivity. The present study evaluated the effect of hydroponic pretreatment of strawberry (*Fragaria x ananassa* cv. ‘Camarosa’) roots with an H_2_S donor, sodium hydrosulfide (NaHS; 100 μM for 48 h), on the response of plants to acute heat shock treatment (42°C, 8 h).

**Results:**

Heat stress-induced phenotypic damage was ameliorated in NaHS-pretreated plants, which managed to preserve higher maximum photochemical PSII quantum yields than stressed plants. Apparent mitigating effects of H_2_S pretreatment were registered regarding oxidative and nitrosative secondary stress, since malondialdehyde (MDA), H_2_O_2_ and nitric oxide (NO) were quantified in lower amounts than in heat-stressed plants. In addition, NaHS pretreatment preserved ascorbate/glutathione homeostasis, as evidenced by lower ASC and GSH pool redox disturbances and enhanced transcription of ASC (*GDH)* and GSH biosynthetic enzymes (*GS, GCS*), 8 h after heat stress imposition. Furthermore, NaHS root pretreatment resulted in induction of gene expression levels of an array of protective molecules, such as enzymatic antioxidants (*cAPX, CAT, MnSOD, GR*), heat shock proteins (*HSP70, HSP80, HSP90*) and aquaporins (*PIP*).

**Conclusion:**

Overall, we propose that H_2_S root pretreatment activates a coordinated network of heat shock defense-related pathways at a transcriptional level and systemically protects strawberry plants from heat shock-induced damage.

## Background

Threats of climate change and global warming render heat stress a general concern for the agricultural sector worldwide [1]. Plants exposed to high temperatures may experience severe cellular injury that may lead to cell death within a short period [2]. The primary targets of heat shock injury in plants are photosynthesis [3], water status [4], carbon assimilation processes [5] and membrane stability [6]. At the cellular level, heat stress results to protein denaturation and aggregation, increased fluidity of membrane lipids, inactivation of enzymes in chloroplast lamella and mitochondria, inhibition of protein synthesis and secondary oxidative stress through the production of reactive oxygen species (ROS) [7].

Consequently, plants manifest different mechanisms for adaptation and protection in elevated temperatures. The initial heat stress signal, probably perceived as the increase of plasmalemma lipid bilayer fluidity [8], triggers downstream signaling processes for transcriptional regulation [9]. Up-regulation of mitogen activated protein kinase (MAPK) transduction pathway through the induction of Ca^2+^ influx [10], ROS signaling and hormonal activation, as well as heat shock protein (HSP)/chaperone signal transduction pathways, seems to be the key players in plant transcriptional regulation under heat stress [11]. As a result, major thermotolerance mechanisms, such as the induction of antioxidant machinery, accumulation of heat shock proteins, osmolytes and secondary metabolite adjustments, are activated, driving to cellular homeostasis and repairing of damaged proteins and membranes [12].

Extensive plant breeding efforts and more recent transgenic approaches have largely validated that heat stress tolerance is a multigenic trait [13]. In addition, benefits from transgenic approaches have been limited and have not led to agronomically improved crops for heat tolerance under field conditions [14]. Thus, considerable attention has been devoted in alleviating the detrimental effects of high temperatures in plants through the exogenous application of various priming agents. Exogenously applied calcium [7], ascorbic acid [15], abscisic acid [16], salicylic acid [17], H_2_O_2_ and NO [18] managed to enhance thermotolerance of treated plants. Furthermore, seed pretreatment with H_2_O_2_ improved heat tolerance of wheat seedlings through the alleviation of oxidative damage and the up-regulation of stress proteins [19].

Sulfur-containing defense compounds (SDCs) are crucial for the survival of plants under biotic and abiotic stress [20]. Recent evidence revealed the role of H_2_S in orchestrating plant responses to environmental stimuli [21,22]. More precisely, exogenous application of H_2_S donor sodium hydrosulfide (NaHS) managed to alleviate heavy metal toxicity in germinating wheat [23], cucumber [24] and barley seedlings [25]. Exogenous application of NaHS was found to promote osmotic stress tolerance in sweet potato [26], soybean seedlings [27] and strawberry plants [28]. Furthermore, H_2_S promoted root organogenesis [29] and was also found to be involved in guard cell signaling [30].

Recent reports showed that NaHS pretreatment significantly increased heat tolerance in tobacco suspension cultured cells [31] and maize seedlings [32,33], respectively. However, whether H_2_S priming could transcriptionally induce a systemic activation of plant defense mechanisms for providing tolerance to subsequent exposure of a heat-sensitive soft fruit crop such as strawberry remains largely unexplored. In the present study we hypothesized that transient pre-exposure of strawberry plant roots to H_2_S may induce systemic thermotolerance to subsequent exposure of plants to heat shock treatment (42°C, 8 h). Therefore, the effects of root pretreatment with H_2_S donor NaHS on several key components of stress tolerance mechanisms in the leaves of strawberry plants were investigated following a combined physiological, biochemical and molecular approach. As far as is known, this is the first study dealing with the employment of H_2_S for the protection of a fruit crop from temperature extremes, as well as the first report to implicate the transcriptional regulation of HSPs and aquaporins in the response.

## Results

### Phenotypic observations

**Figure 1:**
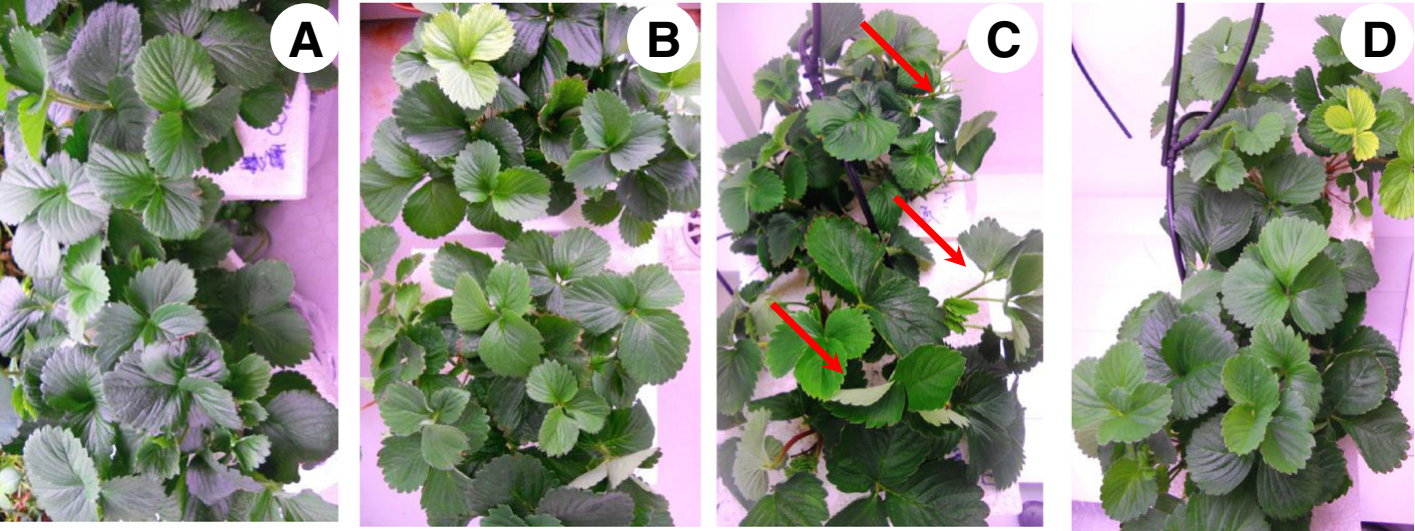
**Phenotypic effects of H**_**2**_**S donor NaHS (100 μM) on strawberry plants exposed to heat shock treatment (42°C) for 8 h.** {**(A)** Control: pretreated with H_2_O and subjected to 23°C for 8 h, **(B)** NaHS: pretreated with NaHS and subjected to 23°C for 8 h, **(C)** Heat: pretreated with H_2_O and subjected to 42°C for 8 h and **(D)** NaHS → Heat: pretreated with NaHS and subjected to 42°C for 8 h}. Red arrows indicate wilted, curled leaves.

Exposure of strawberry plants to 42°C for 8 h resulted in mild wilting and leaf curling (Figure 1C), while NaHS root pretreatment prior to stress exposure exhibited obvious mitigating effect, as evidenced by the conservation of plant leaf turgor and structure (Figure 1D). Non-stressed NaHS-treated plants (Figure 1B) displayed similar phenotype with control plants (Figure 1A), verifying the non-toxic effects of NaHS at the concentration applied.

### Hydrogen sulfide content

**Figure 2:**
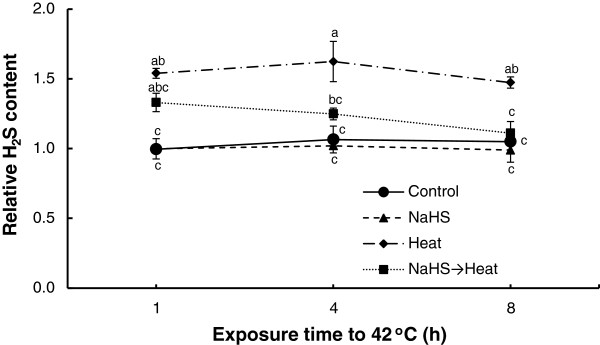
**Effect of H**_**2**_**S donor NaHS (100 μM) on H**_**2**_**S content of strawberry leaves exposed to heat shock treatment (42°C) for 8 h, relatively to content at time point 0 h.** Treatment acronyms are described in Figure 1 caption. Data are means ± SE of three replications. Bars with different letters are significantly different at *p* < 0.05.

Sodium hydrosulfide root pretreatment resulted in significantly higher absolute H_2_S content in strawberry leaves compared with control samples, thus verifying its status as an H_2_S donor (data not shown). Heat stress caused a marked modulation in H_2_S leaf content, manifested by a significant increase after 1, 4 and 8 h of exposure to 42°C compared with control samples. A significant increase in H_2_S content was also recorded in NaHS-pretreated plants after 1 h exposure to heat stress, gradually lowering to control levels thereafter (Figure 2).

### Chlorophyll fluorescence

**Figure 3:**
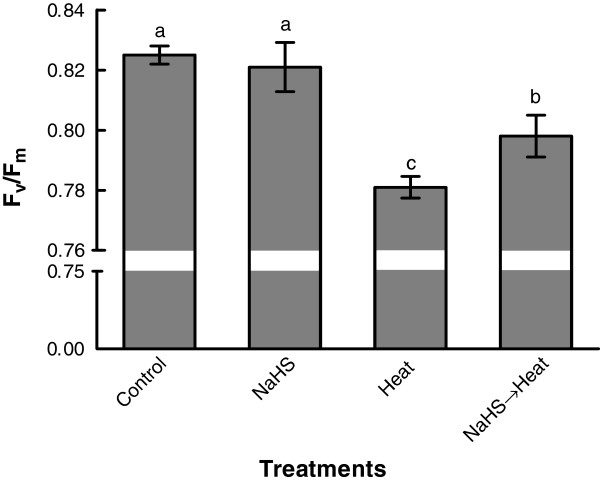
**Effect of H**_**2**_**S donor NaHS (100 μM) on chlorophyll fluorescence (**^**Fv**^**/**_**Fm**_**) of strawberry leaves exposed to 42°C for 8 h.** Treatment acronyms are described in Figure 1 caption. Data are means ± SE of three replications. Bars with different letters are significantly different at *p* < 0.05.

Apparent negative effects of heat stress on F_v_/F_m_ ratio of strawberry plants were registered; a significant reduction on F_v/_F_m_ ratio was measured in plants subjected to heat stress for 8 h. Nevertheless, root pretreatment with NaHS prior to heat exposure enabled strawberry plants subjected for 8 h to heat shock treatment to maintain higher quantum efficiency of photosystem II compared with plants directly exposed to heat stress (Figure 3).

### Cellular damage effects

**Figure 4:**
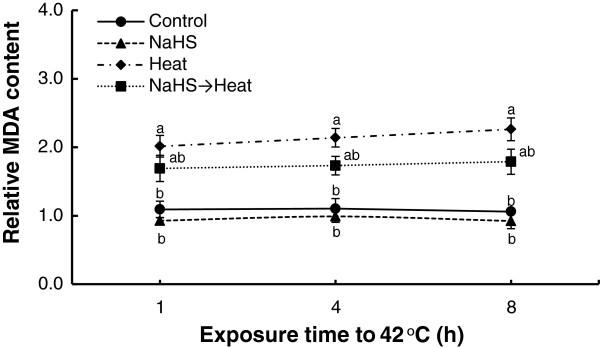
**Effect of H**_**2**_**S donor NaHS (100 μM) on malondialdehyde (MDA) content of strawberry plant leaf tissue exposed to heat shock treatment (42°C) for 8 h, relatively to content at time point 0 h.** Treatment acronyms are described in Figure 1 caption. Data are means ± SE of three replications. Bars with different letters are significantly different at *p* < 0.05.

Heat stress enhanced membrane damage, resulting in increased MDA content (Figure 4). MDA content was doubled within 1 h of exposure to 42°C, following an increasing pattern thereafter. Inversely, NaHS root pretreatment prior to stress exposure managed to mitigate the levels of lipid peroxidation, validated by the lower MDA content compared with non-pretreated stressed plants.

### Hydrogen peroxide and nitric oxide content

**Figure 5:**
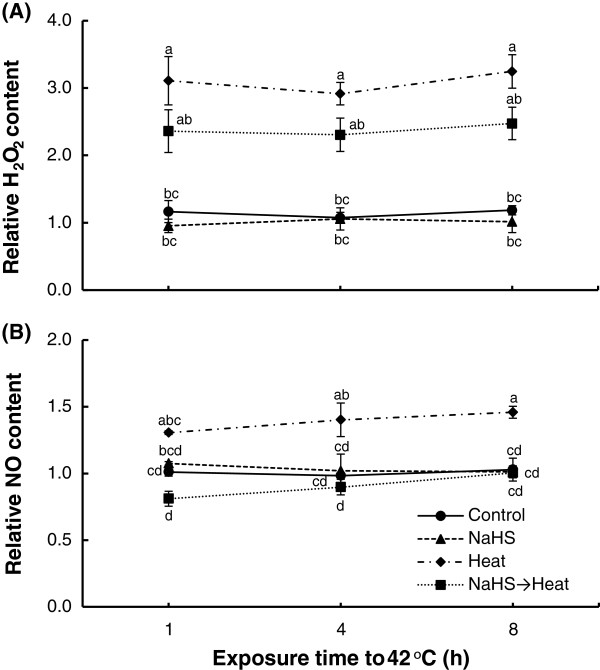
**Effect of H**_**2**_**S donor NaHS (100 μM) on hydrogen peroxide (H**_**2**_**O**_**2**_**) (A) and nitric oxide (NO) (B) content of strawberry plant leaf tissue exposed to heat shock treatment (42°C) for 8 h, relatively to content at time point 0 h.** Treatment acronyms are described in Figure 1 caption. Data are means ± SE of three replications. Bars with different letters are significantly different at *p* < 0.05.

Progressive heat shock caused a marked increase in both H_2_O_2_ and NO content, reaching maximum levels after 8 h (Figure 5). However, lower rates of H_2_O_2_ accumulation were recorded in NaHS-pretreated plants subsequently exposed to progressive heat shock treatment, albeit significantly higher than control, unstressed plants (Figure 5A). In turn, no significant modulation in NO content was recorded in NaHS-treated plants exposed to heat treatment, compared with control samples (Figure 5B).

### ASC and GSH content/redox state

**Figure 6:**
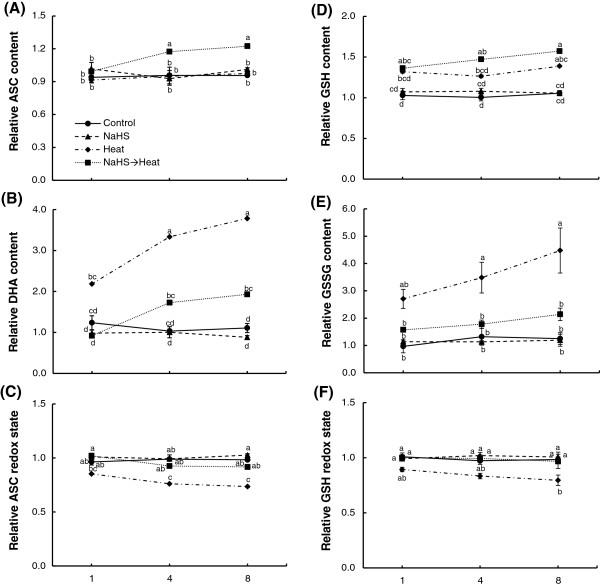
**Effect of H**_**2**_**S donor NaHS (100 μM) on ascorbate and glutathione pool and redox state of strawberry plant leaf tissue exposed to heat shock treatment (42°C) for 8 h, relatively to content at time point 0 h.** {reduced ascorbate (ASC) **(A)**, dehydroascorbate (DHA) **(B)**, ascorbate redox state **(C)**, reduced glutathione (GSH) **(D)**, oxidized glutathione (GSSG) **(E)** and glutathione redox state **(F)**}. Treatment acronyms are described in Figure 1 caption. Data are means ± SE of three replications.

Exposure to elevated temperature for 8 h resulted in a significant increase in both total ascorbate and total glutathione content, while NaHS root pretreatment prior to heat exposure resulted to a greater increase in both antioxidants’ pools. The increase is likely attributed to the increase of both reduced and oxidized forms of ascorbate and glutathione. More precisely, heat stress resulted in non-significant increase in ASC content, while a significant increase in GSH leaf content was recorded. NaHS pretreatment prior to heat exposure resulted in a greater increase of both reduced forms (Figure 6A and D). On the other hand, a substantial increase in both DHA and GSSG content was registered after heat exposure for 8 h. Interestingly, root pretreatment with NaHS managed to ameliorate further oxidation of both antioxidant molecules as evidenced by the lower levels of DHA and GSSG content (Figure 6B and E). Overall, NaHS root pretreatment managed to significantly alleviate both ascorbate and glutathione redox state disturbances compared with non-pretreated stressed plants (Figure 6C and F). In addition, total ascorbate and glutathione pools of NaHS-treated unstressed plants were maintained in similar levels to those observed in control samples.

### Gene expression levels

**Figure 7:**
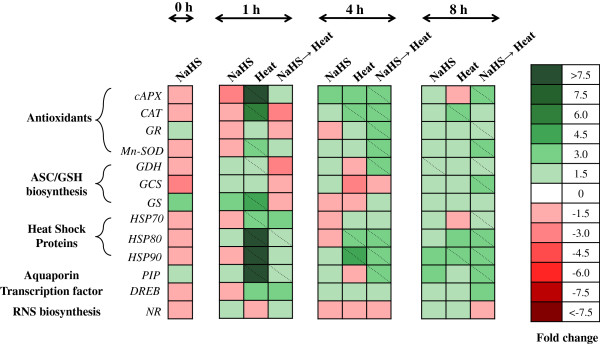
**Heat map showing temporal expression pattern of selected genes associated with enzymatic antioxidants, RNS biosynthesis, redox homeostasis, heat shock proteins and aquaporins in leaves of strawberry plants under non-stress and heat stress conditions.** Following root pre-treatment with 100 μΜ NaHS, the experimental plants were grown either under normal (23°C) or extreme temperature (42°C) conditions for 8 h, as described in Figure 1. Relative mRNA abundance was evaluated by real-time RT-PCR using three biological repeats. Up-regulation is indicated in green; down-regulation is indicated in red. Diagonal dotted lines represent statistically significant values at *p* < 0.05. A scale of color intensity is presented as a legend. Actual relative expression data, obtained from 3 independent replicates, are shown in Additional file 1: Table S1.

The relative expression ratio of a diverse set of specific genes involved in antioxidant machinery, cellular redox regulation, signal transduction and protein structure stability, assayed by quantitative real-time RT-PCR, is presented in Figure 7. These included key enzymatic antioxidant (cytosolic ascorbate peroxidase, *cAPX;* catalase*, CAT;* manganese superoxide dismutase, *MnSOD;* glutathione reductase, *GR*), ascorbate and glutathione biosynthesis (_L_-galactose dehydrogenase, *GDH*; glutamylcysteine synthetase, *GCS;* glutathione synthetase, *GS*), NO biosynthesis (nitrate reductase, *NR*), transcription factor (dehydration-responsive element binding factor, *DREB*), heat shock proteins (*HSP70, HSP80, HSP90*) and aquaporin (*PIP*) genes. Overall, heat stress alone or in combination with NaHS root pretreatment significantly induced mRNA expression levels of most examined genes, being highly dependent on the duration of heat exposure.

The main trends observed were the overall low levels of regulation of all genes examined in plants treated solely with NaHS, compared with control samples, except for *GDH* 8 h after the end of root incubation (Figure 7). In turn, 1 h of heat stress imposition revealed a significant up-regulation of most genes examined (with the exceptions of *GR, NR, GCS* and *DREB*), greatly ameliorated after progressive heat stress exposure. Furthermore, heat treatment caused the rapid accumulation of high levels of *cAPX, PIP, HSP80* and *HSP90* transcripts as early as 1 h after heat stress imposition (see Additional file 1: Table S1). Nevertheless, the expression pattern of most genes examined in non-treated plant suffering heat stress for 8 h was generally similar to respective controls.

Interestingly, the protective effect of NaHS root pre-treatment on heat stress tolerance in terms of transcriptional induction of defense-related genes was recorded 4 h after stress imposition, reaching maximal induction levels for most genes examined (except for *CAT, NR, GS* and *DREB*) after 8 h of heat stress (Figure 7). Conversely, no significant activation of defense-related molecular machinery was recorded 1 h after stress imposition in NaHS-pretreated and subsequently stressed plants.

## Discussion

Heat stress affects a broad spectrum of cellular components and metabolism, often causing irreversible damage to plant growth and development. Despite the constant advances being made towards understanding plant responses to temperatures extremes and improving thermotolerance, examples of plants with heat tolerance through conventional breeding and transgenic approaches are limited and condensed into laboratory conditions [14]. Therefore, compounds that might result in the mitigation of high temperature detrimental effects could potentially be of great importance. We have recently postulated the priming effect of H_2_S in the alleviation of salinity and non-ionic osmotic stress [28]. In the current study, an array of physiological, biochemical and molecular approaches provided novel evidence that root pretreatment with H_2_S donor NaHS enhanced thermotolerance of strawberry plants subsequently exposed to temperature extremes, supporting the notion that H_2_S is a key signaling molecule in plants. This seems to be of pivotal importance for strawberry cultivation, since strawberry growth and productivity are found to be greatly affected by temperature extremes [34]. Furthermore, this study revealed that the role of NaHS in alleviating heat shock stress could be attributed to H_2_S, as the levels of endogenous H_2_S increased following NaHS pretreatment and subsequent stress imposition, in accordance with similar findings by Zhang et al. [27]. Importantly, Zhang et al. [26,29] reported that only H_2_S, and no other Na^+^- or sulfur-containing compounds released from NaHS, have a protective role during abiotic stresses, while similar findings were provided by Li et al. [32] in maize plants under heat stress following an H_2_S inhibitor approach.

Photosystem II (PSII) is the most thermally sensitive component of photosynthesis and its activity is greatly reduced or even partially stopped under high temperatures [35]. Our results are in agreement with previous findings highlighting that maximal photochemical efficiency (F_v_/F_m_) in leaves of several plant species was reduced under heat stress conditions [7,36]. However, NaHS application results in the alleviation of heat stress injuries to PSII activity, as evidenced by the higher F_v_/F_m_ ratio preserved in pre-treated stressed strawberry plants compared with untreated stressed plants (Figure 3).

Furthermore, in order to verify the role of NaHS root pretreatment in alleviating heat shock derived oxidative damage, lipid peroxidation and H_2_O_2_ leaf content were assayed. Results showed that NaHS mitigated heat stress-induced MDA and H_2_O_2_ content increase in strawberry leaves, thus suggesting that oxidative damage to membranes and other cell components was reversed following H_2_S treatment (Figures 4 and 5). Our findings further support previous results showing that NaHS pretreatment resulted in lower content of both MDA and H_2_O_2_ in stressed plants. More precisely, NaHS resulted to the conservation of MDA and H_2_O_2_ content increase against osmotic stress [26,27], or under high aluminum concentration [23]. In addition, we recently provided evidence that NaHS root pretreatment resulted in lower levels of MDA and H_2_O_2_ in strawberry plants suffering ionic and non-ionic osmotic stress [28]. Rapid (within 1 h) accumulation of H_2_O_2_ in heat-stressed plants alone, may suggest a role for H_2_O_2_ in triggering the early expression of heat-shock proteins in stressed plants, as previously reported [37,38]. Our findings are further supported from the transcript levels of major enzymatic antioxidants (*cAPX, CAT, MnSOD, GR*), which were found to be induced in NaHS-pretreated plants in comparison with untreated ones, after 4 and 8 h of exposure at 42°C. Transcript levels are in line with previous observations in NaHS-pretreated strawberry plants exposed to NaCl and PEG-6000 treatment [28], as well as with enhanced antioxidant enzymatic activities in NaHS-treated plants under cadmium [39], heat [32] and drought [27] stress, as well as in NaHS-treated salt-stressed germinating seeds [40], thus rendering the apparent induction of antioxidant enzymes of prime importance for the enhanced tolerance observed.

Besides ROS, NO and other NO-derived products, cumulatively called reactive nitrogen species (RNS), may also be overproduced under abiotic stress conditions, causing secondary nitrosative stress in plants [41,42]. NO has also been shown to act in parallel with other signaling molecules for regulating many biological processes, including responses to abiotic stresses [43-45]. Results indicated that NaHS pretreatment managed to sustain NO content in levels similar to control, as supported by the non-significant modulation of *NR* relative expression compared with control samples. In contrast, strawberry plants experiencing heat shock treatment exhibited a marked increase in leaf NO content, despite similar *NR* expression levels with control samples. Such a negative correlation could be attributed to feedback inhibition of NR [46], possibly due to NO toxicity [47]. However, the increase in NO content in heat-stressed plants may be the key factor driving to the rapid accumulation of *HSP* transcripts observed. Xuan et al. [48] reported that NO acts upstream of AtCaM3 in thermotolerance for the stimulation of DNA-binding activity of heat shock transcription factors and the accumulation of HSPs. Interestingly, recent findings by Li et al. [32] suggested that H_2_S may be a downstream signal molecule in NO-induced heat tolerance of maize seedlings, further supporting the interplay between reactive species towards induced tolerance.

Beside their participation in oxidative processes that may lead to cell damage, ROS participate in redox state-based sensing mechanisms that are activated or amplified in response to environmental stimuli [49,50]. In parallel with ROS detoxification, ascorbate and glutathione are molecules with a regulatory role, since they participate in the redox signaling of the plant cell under abiotic stress conditions [51]. In recent years, several studies have strengthened the notion that high ASC/DHA and/or GSH/GSSG ratios, sustained by increased ASC and GSH or diminution of DHA and GSSG cellular production, may be the key element for enhanced tolerance during abiotic stress exposure [52]. In the current study, we showed that transient root exposure to H_2_S donor NaHS prior to stress exposure managed to sustain higher ratios of ASC/DHA and GSH/GSSG, compared with non-pretreated heat stressed plants, as evidenced by higher ASC and GSH redox states. Heat stress resulted in direct and progressive increase of reduced and oxidized glutathione and oxidized ascorbate. However, NaHS pretreatment prior to heat exposure resulted in an additional increase of reduced ascorbate and glutathione and the diminution of their oxidized forms. The induced expression of *GDH* and *GCS* provides support for these observations, since these enzymes contribute to ASC and GSH biosynthesis and redox homeostasis, respectively. Our results are in agreement with those of Shan et al. [53,54], who reported that the observed induced tolerance in NaHS-pretreated wheat seedlings under water and copper stress was attributed to the increased activity of enzymatic antioxidants and ASC (L-galactono-1,4-lactone dehydrogenase; GalLDH) and GSH biosynthesis (GCS) enzyme activities, as well as to the increased contents of ASC, GSH, total ascorbate and total glutathione, in comparison with untreated stressed seedlings.

Changes in genotypic expression leading to increase synthesis of heat shock proteins (HSPs) is known to be an early and important adaptive strategy in cells that are subjected to all types of stresses [55]. The HSPs, ranging in molecular mass from about 10 to 200 kDa, have been found to accumulate in great amounts during heat stress in various cellular structures, such as cell wall, chloroplasts, ribosomes and mitochondria [2,56]. Their role in maintaining cellular homeostasis under heat stress, mainly by assisting the correct folding of stress-accumulated misfolded proteins, preventing their aggregation and promoting proteolytic degradation of misfolded or denatured proteins, as well as in participating in signal transduction, has been recently reviewed [57]. In the current study, HSPs appeared to be substantially accumulated in cells immediately after heat stress exposure, since *HSP70*, *HSP80* and *HSP90* expression levels were found to be significantly induced, compared with control samples, as early as after 1 h of exposure to 42°C. The increased expression levels of the examined *HSPs* may be attributed to the rapid accumulation of H_2_O_2_ and NO, which function as signaling molecules for the production of HSPs, as also previously reported [37,38,48]. Induction of *HSPs* in stressed plants was lowered after prolonged exposure to heat treatment. Our results are in agreement with previously reported findings, based on transcriptomic and proteomic analyses, highlighting the importance of the early accumulation of HSPs for the acquisition of thermotolerance in plants experiencing temperature extremes [57,58]. The role of HSP70 in *Arabidopsis thaliana* heat shock responses and thermotolerance has recently been elucidated [59]. On the contrary, NaHS treatment prior to heat stress induced the up-regulation of HSPs only after 4 h of exposure to heat shock conditions, providing evidence for their possible contribution to the observed mitigation of heat stress devastating effects, made apparent after 4 h of exposure to 42°C. The lower H_2_O_2_ and NO contents during the early stages of heat exposure in NaHS-pretreated plants can be attributed to late accumulation of HSPs (after 4 h of stress imposition), since both active molecules function in parallel with HSPs biosynthesis. In turn, acclimation of *Aloe vera* plants to less severe temperature extremes resulted in elevated expression of *HSPs*[60].

The molecular and functional characterization of aquaporins (PIPs), a class of membrane proteins that facilitate water diffusion across cell membranes, has revealed the significance of their regulation in response to adverse environmental stimuli [61,62]. In plants, aquaporins are localized in abundance in the plasmalemma and the vacuolar membrane [63]. Recent studies shed light to the possible role of aquaporins in abiotic stress tolerance. Ayadi et al. [62] confirmed the role of PIP1 and PIP2 in osmotic and salinity stress tolerance in durum wheat. Furthermore, Iglesias-Acosta et al. [64] reported a decline in *PIP1* and *PIP2* transcript abundance in the roots of broccoli plants under increasing temperature, while Chen and Arora [65] highlighted the role of aquaporins (*SoPIP2;1 and SoδTIP*) during the recovery of spinach leaves from reversible freeze-thaw injury. In the current study, gene expression analysis revealed that *PIP* had the same expression pattern as *HSPs*, suggesting that heat exposure caused the early induction (after 1 h) of *PIP* expression, which was eliminated after prolonged heat stress. On the other hand, NaHS pretreatment-induced *PIP* up-regulation was apparent after 4 h exposure at 42°C (see Additional file 1: Table S1).

## Conclusion

A coordinated, transient induction of antioxidants, HSPs and aquaporin gene expression was registered when plants were exposed to heat shock treatment, which was de-escalated as stress imposition progressed. The early (1 h) and transient up-regulation of defense-related genes in plants exposed directly to heat stress conditions seems to provide inadequate signal for transcriptional regulation of defense pathways, leading to weak heat stress responses. On the contrary, NaHS root priming demonstrated a ‘delayed’ (after 4 h) but prolonged (maximized after 8 h) transcriptional activation of defense responses, resulting in acquired thermotolerance via the sufficient production of protective molecules such as HSPs and antioxidants. The energy-consuming coordinated orchestration of several independent pathways is most likely feasible through increased photosynthetic capacity in NaHS-treated plants [66]. Overall, data reported herein provide novel information for the improvement of crop tolerance to heat stress and lends additional support to the suggested role of H_2_S in plant responses to environmental stimuli. The current state of knowledge in defining the contribution of H_2_S in plant tolerance mechanisms to abiotic stress warrants further investigation, including the potential application of synthetic inhibitors of H_2_S biosynthesis (e.g [67]).

## Methods

### Plant growth and stress treatments

Forty-eight strawberry (cv. ‘Camarosa’) plants were grown in peat in greenhouse for six months and subsequently transferred and grown hydroponically in continuously aerated half-strength Hoagland nutrient solution in a growth room with 16 h photoperiod (250 μmol m^-2^ s^-1^), 23°C/20°C day/night temperature and 65% relative humidity. After one week, roots of one half of the plants were incubated in deionized water containing an H_2_S donor, sodium hydrosulfide (NaHS; 100 μM for 48 h; changed every 12 h). At the end of the incubation period, plants were transferred to half-strength Hoagland nutrient solution. As a result, plants either pretreated or not with NaHS and grown hydroponically in continuously aerated half-strength Hoagland nutrient solution were simultaneously exposed (0 h, stress imposition) or not to elevated temperature treatment (42°C) for 8 h. Overall, strawberry plants were subjected to 4 treatments, as presented in detail in Figure 1 and described schematically in Additional file 2: Figure S1. Each treatment was independently run in triplicate, and each replicate consisted of 4 individual plants. Fully expanded leaves were sampled immediately after the imposition of heat stress treatment (0 h) and after 1, 4 and 8 h of exposure to 42°C. Leaves were flash-frozen in liquid nitrogen and stored at -80°C, unless otherwise stated.

### Physiological and biochemical measurements

The ratio of variable fluorescence to maximum fluorescence (F_v/_F_m_), representing the maximum photochemical efficiency of photosystem II (PSII), was determined using a portable chlorophyll fluorometer (OptiSci OS-30p Chlorophyll Fluorometer, Opti-Sciences Inc, USA). Leaves were incubated in dark for 1 h prior to measurements. The comparative rates of lipid peroxidation in strawberry leaves were assayed in terms of MDA content according to Heath and Packer [68].

### Reactive species quantification

Nitric oxide content was indirectly assayed by measuring nitrite (NO_2_^–^), a stable and non-volatile breakdown product of NO reduction, via the Griess reaction, as described by Zhou et al. [69]. Leaf H_2_O_2_ content was assayed as described by Loreto and Velikova [70], while H_2_S content was determined following the methodology described by Nashef et al. [71]. Descriptions of all reactive species quantification protocols followed can be found in [28].

### ASC and GSH content/redox state

Reduced ascorbate (ASC) and dehydroascorbate (oxidized ascorbate; DHA) were measured according to Foyer et al. [72]. Redox state of ascorbate was expressed as the ratio of ASC to total ascorbate (ASC/ASC + DHA). The levels of reduced glutathione (GSH) and oxidized glutathione (GSSG) were assayed as described by Griffith [73], while the glutathione redox state was expressed as the ratio of GSH to total glutathione (GSH/GSH + GSSG).

### RNA isolation, cDNA synthesis and gene expression analysis

Total RNA from strawberry leaves was isolated following the protocol described by [74]. The integrity of total RNA was checked spectrophotometrically (A260/A280) using a NanoDrop Spectrophotometer ND-1000 (Labtech International Ltd, Rigmer, UK), followed by gel electrophoresis. For first strand cDNA synthesis, 1 μg of total RNA was reverse-transcribed using the Primescript 1^st^ Strand Synthesis kit, according to manufacturer’s instructions (Takara Bio Inc., Japan). Quantitative real-time RT-PCR was performed in a final volume of 10 μl, containing 4 μl of ten-fold diluted first strand cDNA, 0.5 μl of each of the gene specific primers (10 pM) and 5 μl of KAPA SYBR^®^ FAST qPCR supermix (Takara Bio Inc). The initial denaturation stage was at 95°C for 3 min, followed by 40 cycles of amplification (95°C for 30 s, Ta°C for 45 s, and 72°C for 45 s) and a final elongation stage at 72°C for 5 min. Gene amplification cycle was followed by a melting curve run, carrying out 61 cycles with 0.5°C increment between 65°C - 95°C. PCR reactions of each treatment were performed in triplicate with an iQ5 real-time PCR detection system (Bio-Rad Laboratories, Inc., California, USA). Fold change in RNA expression was estimated using threshold cycles. The housekeeping reference gene used was *18S* (Ta = 46°C) [75]. The statistical analysis of qRT-PCR results (pairwise fixed reallocation randomization test) was performed using the REST software, according to Pfaffl et al. [76]. The list of gene-specific primers used is presented in Additional file 3: Table S2.

### Statistical analysis

Statistical analysis was carried out using the software package SPSS v17.0 (SPSS Inc., Chicago, USA) and the comparison of averages of each treatment was based on the analysis of variance (One-Way ANOVA) according to Duncan’s multiple range test at significance level 5% (*P ≤ 0.05*).

## Abbreviations

cAPX: Cytosolic ascorbate peroxidase; ASC: Reduced ascorbate; CAT: Catalase; DREB: Dehydration-responsive element binding factor; GCS: Glutamylcysteine synthetase; GDH: L-galactose dehydrogenase; GR: Glutathione reductase; GS: Glutathione synthetase; GSH: Reduced glutathione; H2S: Hydrogen sulfide; HSPs: Heat shock proteins; MnSOD: Manganese superoxide dismutase; NaHS: Sodium hydrosulfide; NO: Nitric oxide; NR: Nitrate reductase; PIP: Aquaporin; RNS: Reactive nitrogen species; ROS: Reactive oxygen species.

## Competing interests

The authors declare that they have no competing interests.

## Authors’ contributions

AC, GAM and VF designed the study, while VF also designed the oligonucleotide primers used herein. AC performed the experiments and took care of the plants. AC and PF carried out the laboratory work and data analysis. AC, GAM and VF wrote the manuscript and prepared the figures. All authors read and approved the manuscript.

## Supplementary Material

Additional file 1: Table S1 Effects of H_2_S donor NaHS on the relative mRNA expression (fold change) of enzymatic antioxidants, heat shock proteins, aquaporins and enzymes involved in RNS biosynthesis, redox homeostasis and transcription regulation, in leaves of strawberry plants under non-stress and heat shock conditions compared with controls, as determined by qRT-PCR. Click here for file

Additional file 2: Figure S1 Schematic representation of the experimental design. Click here for file

Additional file 3: Table S2 Oligonucleotides used as primers for real-time RT-PCR. Click here for file
