# Supplementary material for: Sodium hydrosulfide induces systemic thermotolerance to strawberry plants through transcriptional regulation of heat shock proteins and aquaporin
Source: BMC Plant Biol. 2014 Feb 5;14:42. doi: 10.1186/1471-2229-14-42 (PMC3933230; doi:10.1186/1471-2229-14-42)
Supplement: Additional file 1: Table S1 — Effects of H2S donor NaHS on the relative mRNA expression (fold change) of enzymatic antioxidants, heat shock proteins, aquaporins and enzymes involved in RNS biosynthesis, redox homeostasis and transcription regulation, in leaves of strawberry plants under non-stress and heat shock conditions compared with controls, as determined by qRT-PCR. [file 1471-2229-14-42-S1.doc]

Additional file 1: Table S1. Effects of H_2_S donor NaHS on the relative mRNA expression (fold change) of enzymatic antioxidants, heat shock proteins, aquaporins and of enzymes involved in RNS biosynthesis, redox homeostasis and transcription regulation, in leaves of strawberry plants under non-stress and heat shock conditions compared with controls, as determined by qRT-PCR. Following root pre-treatments with NaHS, plants were exposed or not to heat shock treatment (42 ^o^C) for 8 h, as described in Figure 1. Tissues were sampled immediately after NaHS pre-treatment (just before stress imposition; 0 h) and 1, 4 and 8 h after stress imposition. Values in bold letters denote statistically different values according to pairwise fixed reallocation randomisation test (*P < 0.05*). Values in green represent significantly induced samples, while non-colored boxes represent samples with expression pattern not significantly changed, compared with control samples. Higher green color intensity represented higher fold of expression induction. *18S* rRNA was used as a housekeeping gene control.
