# Supplementary material for: Sodium hydrosulfide induces systemic thermotolerance to strawberry plants through transcriptional regulation of heat shock proteins and aquaporin
Source: BMC Plant Biol. 2014 Feb 5;14:42. doi: 10.1186/1471-2229-14-42 (PMC3933230; doi:10.1186/1471-2229-14-42)
Supplement: Additional file 2: Figure S1 — Schematic representation of the experimental design. [file 1471-2229-14-42-S2.doc]

**Additional file 2: Figure S1.** Schematic representation of the experimental design. Treatments are as follow: **Control:** pretreated with H_2_O and subjected to 23 ^o^C, **NaHS:** pretreated with NaHS and subjected to 23 ^o^C, **Heat:** pretreated with H_2_O and subjected to 42 ^o^C for 8 h and **NaHS→Heat:** pretreated with NaHS and subjected to 42 ^o^C for 8 h.
