# Supplementary material for: Sodium hydrosulfide induces systemic thermotolerance to strawberry plants through transcriptional regulation of heat shock proteins and aquaporin
Source: BMC Plant Biol. 2014 Feb 5;14:42. doi: 10.1186/1471-2229-14-42 (PMC3933230; doi:10.1186/1471-2229-14-42)
Supplement: Additional file 3: Table S2 — Oligonucleotides used as primers for real-time RT-PCR. [file 1471-2229-14-42-S3.doc]

Additional file 3: Table S2. Oligonucleotides used as primers for real-time RT-PCR.

| Gene | Primer | Primer sequence | Reference | Τm (^o^C) |
| --- | --- | --- | --- | --- |
| *18S* | For  Rev | 5-ACC GTA GTA ATT CTA GAG CT-3  5-CCA CTA TCC TAC CAT CGA AA-3 | Bustamante et al.  (2006) | 46 |
| *GS* | For  Rev | 5΄-GGG ACG AAA AAG GTT CAA CA-3΄  5΄-GCA TCA GGA TGT AAG CAG CA-3΄ | Christou et al.  (2013) | 56 |
| *GCS* | For  Rev | 5΄-ACA GGC TCG TCC TAC TGC AT-3΄  5΄-TCC TAC ACC TGC ATT GTC CA-3΄ | Christou et al.  (2013) | 56 |
| *GDH* | For  Rev | 5΄-CAA GGT CGA GCT TCG AGA AC-3΄  5΄-CAA GCA CCT TCT CCG ACA AT-3΄ | Christou et al.  (2013) | 56 |
| *GR* | For  Rev | 5΄-AGC ATG ACT GGA GCA CAT TG-3΄  5΄-AGG CAA ATC AAG AGC AGC AT-3΄ | Christou et al.  (2013) | 58 |
| *CAT* | For  Rev | 5΄-CAC CTG TCA TTG TGC GTT TC-3΄  5-CTT TCT GGA TGG TGG GAA AA-3΄ | Christou et al.  (2013) | 56 |
| *cAPX* | For  Rev | 5΄-CAC AAG GAA CGG TCT GGA TT-3΄  5΄-CGC AGC GTA TTT CTC AAC AA-3΄ | Christou et al.  (2013) | 56 |
| *MnSOD* | For  Rev | 5΄-TGT GGC TGG CTT TAG ACA AA-3΄  5΄-CTT CCA ATT GAT GAC CTT CCA-3΄ | Christou et al.  (2013) | 56 |
| *NR* | For  Rev | 5΄-AGG GAA ACA CCC CTT CAA CT-3΄  5΄-TGC TTC ACC ATG TTC TGC TC-3΄ | Christou et al.  (2013) | 56 |
| *DREB* | For  Rev | 5΄-CGG AGT TGG TTT TCC AAG AA-3΄  5΄-TCC GGG TAC TCG TTC AAA TC-3΄ | Christou et al.  (2013) | 56 |
| *HSP70* | For  Rev | 5΄-CAC CAC TGT CAC TGG GTT TG-3΄  5΄-TCT CCC CTT GGC GAA CAT TG-3΄ | TA2408 57918* | 56 |
| *HSP80* | For  Rev | 5΄-CCA GGC TTT GAG GGA TAA CA-3΄  5΄-GAT CCT GTT GCC AAA GGT GT-3΄ | CO817794* | 56 |
| *HSP90* | For  Rev | 5΄-TTG GGT GAT CGT GTT GAC AA-3΄  5΄-GTC TGC ATC AGC CCT CTT TC-3΄ | TA3063 57918* | 56 |
| *PIP* | For  Rev | 5΄-GCT GTT CTT GGC TCG TAA GG-3΄  5΄-ACA CAG CAA ACC CAA TAG GC-3΄ | CX661168* | 56 |

^*^ *Fragaria* EST sequence Accession no.
